# Increasing Functionality of Fish Leather by Chemical Surface Modifications

**DOI:** 10.3390/polym15193904

**Published:** 2023-09-27

**Authors:** Achiad Zilberfarb, Gali Cohen, Elizabeth Amir

**Affiliations:** Department of Polymer Materials Engineering, Shenkar College of Engineering and Design, Anna Frank 12, Ramat Gan 5252626, Israel

**Keywords:** fish leather, surface treatment, leather finishing, polyaniline, hydrophobic coating

## Abstract

Fish skin is a by-product of the fishing industry, which has become a significant environmental pollutant in recent years. Therefore, there is an emerging interest in developing novel technologies to utilize fish skin as a versatile raw material for the clothing and biomedical industries. Most research on finishing procedures is conducted on cattle leather, and practically very limited information on fish leather finishing is found in the literature. We have developed three functional surface finishing treatments on chromium (CL)- and vegetable (VL)- tanned salmon leather. These treatments include hydrophobic, oil repellent, and electro-conductive ones. The hydroxyl functional groups present on the surface of the leather were covalently grafted with bi-functional aliphatic small molecule, 10-undecenoylchloride (UC), by esterification reaction forming hydrophobic coating. The surface hydrophobicity was further increased via covalent binding of perfluorodecanethiol (PFDT) to the double bond end-groups of the UC-modified leather via thiol-ene click chemistry conditions. The oleophobic coating was successfully developed using synthesized fluorinated silica nanoparticles (FSN) and polyvinylidene fluoride-co-hexafluoropropylene (PVDF-HFP), showing oil repellency with a contact angle of about 100° for soybean oil and n-hexadecane. The electrically conductive coating was realized by the incorporation of conjugated polymer, polyaniline (PANI), via in situ polymerization method. The treated leather exhibited surface resistivity of about 5.2 (Log (Ω/square)), much lower than untreated leather with a resistivity of 11.4 (Log (Ω/square)).

## 1. Introduction

As the population of the world increases, aquaculture usage is constantly growing [1]. About 120 million tons of fish has been captured yearly for utilization in the food industry [2]. However, about 50% of the fish weight is considered waste, including protein-rich parts such as the scales, bones, and skin. The fish skin is usually thrown away but can be potentially utilized for the fashion and other industries, similar to other exotic animals such as snakes [3,4,5,6]. For the fish skin to become a leather, a tanning process should be applied, which fixes the collagen fibers in the leather matrix using tannin-based reagents. Chromium tanning is the common tanning process, providing the best leather properties in the shortest processing time [7]. Despite that, a great effort is turned to replacing chromium-based reagents which are harmful to the environment. This replacement is often carried out using a vegetable tanning process involving more natural and environmentally friendly ingredients [8]. Surface coating and finishing is also an important stage in the fabrication process of leathers since, in this stage, the leather gains some of its most important characteristics, such as water and oil repellence, water vapor permeability, and wet and dry fastness [9,10,11]. Leather hydrophobicity is necessary to prevent the unpleasant feeling when wearing wet leather and prevent bacteria growth [12,13], which can be potentially used to develop antibacterial leather [14,15]. Oil repellence is also an important finishing property that prevents the staining of the leather by greasy materials [16]. Both properties can be achieved by careful design and modification of surface free energy and surface roughness [9,12,17,18,19]. Three main strategies to achieve waterproofing were reported in the literature for cattle leather. The first and the most traditional is filling and closing the space between the fibers with water-insoluble substances such as solid grease or molten wax. This method is known as fat liquoring [20,21,22,23]. Fat liquoring can give a unique look and feel similar to leather but also makes it heavy and completely blocks any passage of gasses through the leather [22].

The second and the most common strategy is to coat the leather with a thin polymeric film that will seal the leather and prevent water penetration. The most useful polymers in this method are acrylates [24,25,26] as they combine the necessary properties such as good film forming, flexibility, high gloss, and various commercially available monomers such as butyl methacrylate and methyl methacrylate [13]. Polyacrylate can be used as nanocomposite coating for leather [27,28,29] to improve durability with the incorporation of nano clay nano-silica particles [10,30,31] and other functionalities such as an antibacterial effect using ZnO or Ag nanoparticles [14,32]. The polyurethane family is also used for leather coatings [33] to achieve waterproofing [34] or antibacterial effects [35]. The electrical conductivity of the leather surface holds great promise in smart textile applications such as wearable electronics [36], human motion detection sensors [37], and electromagnetic shielding leather [38], which can be obtained by carbon nanotubes [39] and electro-conductive conjugated polymeric coatings such as PANI [40,41] and polypyrrole [42,43,44]. Although coating the leather with a thin polymeric film can improve its properties and essential functionalities, this method has one crucial setback. The water vapor permeability is greatly reduced, a significant property that affects the comfortable feeling when wearing the leather. The third approach involves chemical grafting of the functional groups of the fibers with low surface energy molecules [18,45,46]. In this approach, the hydrophilic sites are covered and cannot absorb the water molecules, however, gas molecules can penetrate through the leather due to the space between the fibers remaining unsealed [47].

Most of the reported finishing and coating studies are based on cattle hides and to the best of our knowledge, synthetic strategies on fish leather surface coatings have not been reported so far. Herein, we developed three efficient fish leather finishing methods including hydrophobic, oleophobic and electro-conductive ones. The surface modifications were conducted on CL and VL to investigate if there is a difference in the finishing quality of the two tanned leathers due to the different chemical compositions of the tanning agents. The hydrophobic treatment was achieved by an esterification between the hydroxyl moieties present on the leather surface and the acyl chloride group in UC [48]. Additional covalent surface grafting was carried out utilizing the double bond end-groups on the UC-modified leather and thiol-containing PFDT under thiol-ene click reaction conditions [49]. The oleophobic coating was obtained by synthesized FSN and PVDF-HFP coating solutions. Lastly, the electro-conductive coating was introduced using PANI via situ oxidative polymerization of the aniline monomer.

## 2. Material and Methods

### 2.1. Materials

VL and CL were received from Nordic Fish Leather Company (Sauðárkróki, Iceland). VL tanning was carried out using Mimosa extract. UC was purchased from Tzamal D-Chem (Petah Tikva, Israel). Aniline (99.0%), ammonium persulfate (98.0%) (APS), 2,2-dimethoxy-2-phenylacetophenone (DMPA), PFDT, PVDF-HFP, tetraethyl orthosilicate (TEOS), and 1H,1H,2H,2H-perfluorodecyltriethoxysilane (FAS) were purchased from Sigma Aldrich (St. Louis, MO, USA). Triethylamine (TEA), chloroform, dichloromethane, dimethylformamide (DMF), ethanol (EtOH), and hydrochloric acid (HCl) were purchased from Bio-Lab Ltd. (Jerusalem, Israel).

### 2.2. Surface Modification of Leather with UC

Hydrophobic surface modification was carried out according to our previously published procedure [48]. VL- and CL-tanned salmon leathers (25 cm^2^) were immersed in 50 mL chloroform solution containing 10.6 mL TEA (0.076 mol, 1.1 equiv), followed by the addition of 15 mL UC (0.069 mol, 1 equiv). The reaction was carried out at room temperature overnight, followed by successive rinsing of the treated leathers with water, ethanol, and dichloromethane. Finally, the leathers were subjected to Soxhlet extraction for 6 h using ethanol and were then dried in a vacuum oven at 45 °C overnight.

### 2.3. Functionalization of Leather Surface Using PFDT

PFDT was covalently grafted to the surface of UC-modified fish leathers following our previously reported procedure [49]. UC-modified leather samples (6.25 cm^2^) were immersed in 5 mL chloroform solution containing 0.25 mL PFDT (0.87 µmol, 1 equiv) and 0.02 g DMPA (0.08 µmol, 0.09 equiv) in a sealable vessel. Then, the vessel was irradiated at 365 nm for 20 min on one side of the leather. The leather was then rinsed with dichloromethane, water, and ethanol followed by Soxhlet extraction for 3 h using dichloromethane and then dried in a vacuum oven at 45 °C for 3 h.

### 2.4. PANI Coating of Leather

The coating of the fish leathers with PANI was carried out via in-situ polymerization of aniline according to our previously published method [50]. CL and VL samples (100 cm^2^) were immersed in 160 mL HCl 1M solution containing 3.44 mL aniline (0.038 mol) and mixed using an orbital shaker for 5 min (70 rpm). Then, 40 mL of HCl solution containing 10.7 g of APS (0.046 mol) was introduced to the vessel, and the polymerization was carried out for 30 min. The coated leathers were washed with water and ethanol, and dried overnight.

### 2.5. PVDF-HFP/Nano-Silica Particle Coating Leather

The preparation of the FSN solution was carried out as reported in the previous method with some modifications [51]. First, 4 mL ammonium hydroxide and 50 mL ethanol were mixed, followed by the addition 4.5 mL TEOS. The solution was stirred for 2 h, followed by the addition of 0.25 mL (1.7 μmol) FAS. The reaction mixture was stirred for another 1 h at room temperature to form a hydrophobic silica particulate. PVDF-HFP/FAS solution was prepared by combining 2.5 g PVDF-HFP and 0.5 mL FAS in 50 mL DMF and stirred for 30 min. A two-step dip-coating process was used for leather coating. First, to attach the silica nanoparticles to the surface, the leather was immersed into the silica particulate solution for 30 min, followed by drying at room temperature for 20 min without rinsing. Next, the particle-coated leather was immersed into the polymeric solution for 30 min to produce a surface coating containing PVDF-HFP/FAS. Finally, the modified leather was dried at 80 °C for 1 h.

### 2.6. Characterization Methods

Water contact angle measurements. A contact angle analyzer (OCA20, DataPhysics, Riverside, CA, USA) was used to examine the hydrophobic surfaces properties of the leathers. Water contact angle (WCA) values are reported as an average of ten measurements on each leather sample using 5 μL Millipore water droplets.

Fourier transform infrared spectroscopy. A Bruker Alpha-P Fourier transform infrared (FTIR) spectrometer with an attenuated total reflectance (ATR-IR) crystal was used to produce the spectra of the fish leather samples within the range of 400–4000 cm^−1^.

Scanning electron microscopy. Scanning electron microscopy (SEM) images were produced using a JSM-IT200 microscope (JEOL, Akishima, Tokyo).

Resistivity measurements. Surface resistivity measurements were conducted using resistivity test fixture apparatus (KEITHLEY, Tektronix, OH, USA). The leather samples (10 cm × 10 cm) were placed between two stainless steel electrodes, and an electric current was applied (5 Volt, 0.5 mA).

X-ray photoelectron spectroscopy. X-ray photoelectron spectroscopy (XPS) measurements were performed on a Kratos Axis Ultra X-ray photoelectron spectrometer (Kratos Analytical Ltd., Stretford, UK), using an Al Kα monochromatic radiation source (1486.7 eV) with a 90° take-off angle (normal to analyzer). The vacuum pressure in the analyzing chamber was maintained at 2 × 10^−9^ Torr. The binding energies were calibrated relative to the C 1s peak energy position as 285.0 eV. Casa XPS (Casa Software Ltd.) and the vision data processing program (Kratos Analytical Ltd.) were used for data analysis.

Atomic force microscopy. Atomic forced microscopy (AFM) was used to provide topographic images of the leathers. Surface morphology was studied using an Innova AFM mounted on an active anti-vibration Table with a “Bruker” non-contact silicon probe (micro-fabricated Si oxide RTESP Ultra-sharp with integrated pyramidal tips). The 512 × 512 pixel images were collected using tapping mode with a scan size of up to 10 µm at a scan rate of 0.5 Hz. The root means square roughness, which is the deviation in the surface from the mean plane, was used to quantify the leather surface roughness.

Water vapor permeability measurements. Determination of water vapor permeability (WVTR) was carried out according to ISO 14268:2012 [52]. Mocon Permatran-w (model 3/34) instrument was used to compare water vapor permeability between neat and coated leathers.

## 3. Results and Discussion 

### 3.1. Incorporation of Electro-Conductive Coating

**Scheme 1:**
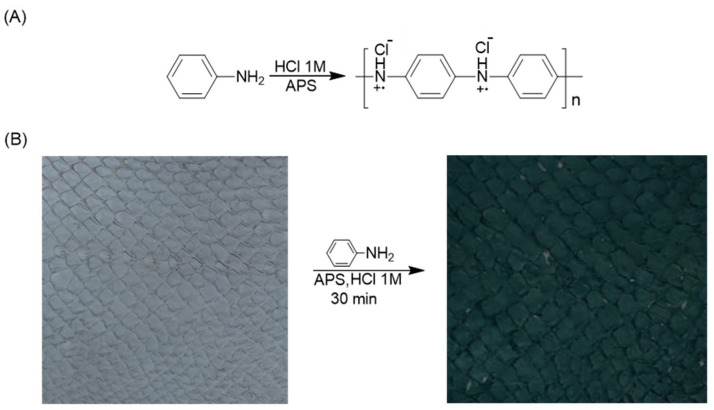
(**A**) Polymerization reaction of aniline monomer to form PANI. (**B**) Surface coating of leather (samples of 10 × 10 cm) with PANI.

Polyaniline was chosen for the fabrication of electro-conductive coating on fish leather due to its excellent electrical conductivity, flexibility, light weight, and ease of preparation using an aqueous solution. Due to its unique chemical structure containing conjugated double bonds, PANI coatings on textiles and other flexible substrates were shown to increase the electrical conductivity by several orders of magnitude [53]. In the present study, in-situ polymerization of aniline monomer using APS as an oxidative reagent in an acidic environment yielded the electro-conducting coating on fish leather (Scheme 1A). After 30 min of polymerization, a distinctive dark green color appeared on the surface of the leather, indicating the presence of PANI emeraldine salt, as shown in Scheme 1B.

**Figure 1:**
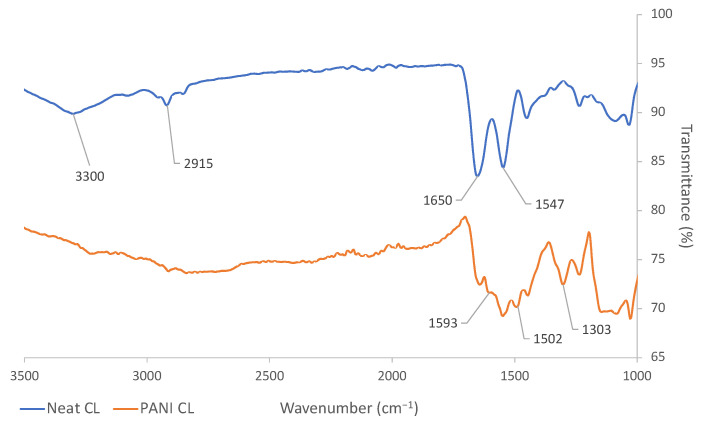
ATR-IR results of neat and PANI-coated CL.

An ATR-IR spectroscopic characterization method was employed to validate the formation of PANI after the polymerization process. ATR-IR spectra are shown in Figure 1 for neat and PANI coated CL, confirming the presence of PANI on the surface of the leather. In the spectra of neat CL, the peaks attributed to the general structure of collagen included the ones at 1547 cm^−1^, which was attributed to the primary amine groups; 1650 cm^−1^, ascribed to the carbonyl stretching; 2915 cm^−1^, associated with the methylene asymmetrical stretching, and the broad peak at 3300 cm^−1^ corresponding to both secondary amine and hydroxyl groups [54,55]. For PANI-treated CL, a characteristic peak at 1303 cm^−1^ was assigned to the carbon-nitrogen bond stretching of the secondary amine group of the PANI backbone. The peaks at 1502 and 1593 cm^−1^ were attributed to the C=C stretching of the benzenoid and quinoid moieties, respectively. Similar results were also observed for PANI-coated VL and presented in Figure S1 in the Supplementary Materials.

**Figure 2:**
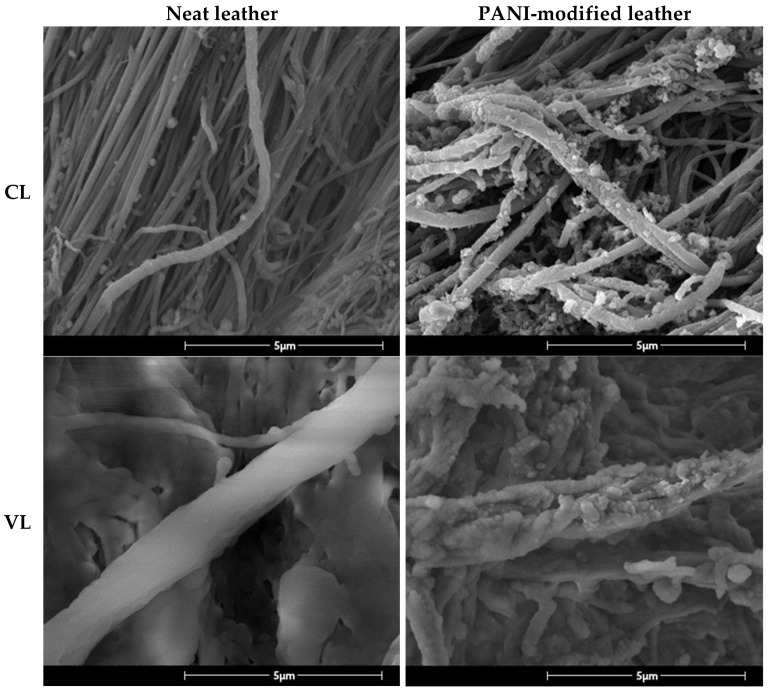
SEM photographs showing the morphology of CL and VL before and after PANI coating.

The morphology of the PANI coating on the surface of CL and VL was further studied using SEM (Figure 2). The SEM images show clear differences in surface morphology between neat and PANI-coated leathers. As a result of the coating process, the surface morphology of the collagen fibrils was changed from smooth to a rougher one due to the formation of PANI aggregates. Furthermore, while an average diameter of collagen fibrils of neat leathers was 0.19 µm, it was increased to about 0.28 µm for PANI coated leathers (Figure S5 and Table S1). This increase in the fibril thickness indicates the formation of PANI coating with a thickness of about 100 nm. The obtained results are in good agreement with the ones previously reported for PANI coated viscose fabric produced using similar method [55]. 

To evaluate the electronic properties of the PANI-coated fish leathers, surface resistivity measurements were carried out. The results of the resistivity measurements revealed that the PANI coating had a significant effect on the surface resistivity of both types of fish leather, transforming the leathers from insulating into semi-conducting ones. Both scale and flesh sides of PANI-coated CL exhibited surface resistivity of about 5 (Log (Ω/square)), which was six orders of magnitude lower in comparison with the untreated leather (Table 1). Similarly, the surface resistivity of PANI-coated VL on the scale side was reduced from 11.7 (Log (Ω/square)) to 6.8 (Log (Ω/square)), and from 11.7 (Log (Ω/square)) to 5.2 (Log (Ω/square)) on the flesh side. Moreover, low standard deviation values were obtained for all tested leathers, indicating that the coating process was uniform and reproducible (Table 1). A reduction in surface electrical resistivity in the range of several orders of magnitude was also observed in our previous study on PANI-coated polyester and viscose non-woven fabrics [50] and polypyrrole coated leather reported by Pfleger et al. [56].

### 3.2. Hydrophobic Surface Treatment

**Figure 3:**
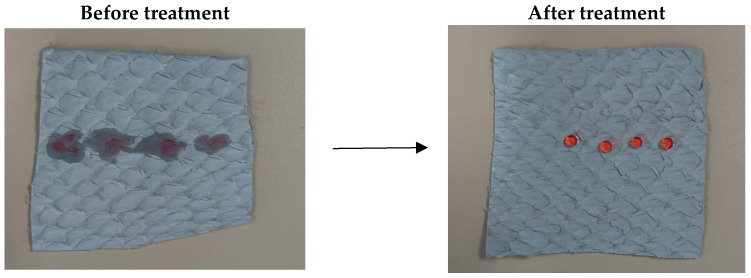
Photographs (samples of 5 × 5 cm) of CL before and after UC treatment taken using drops of water (dyed red).

**Scheme 2:**
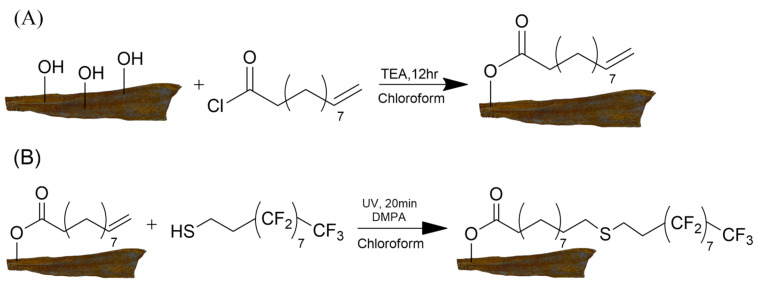
(**A**) Surface covalent modification of leather with UC. (**B**) Functionalization of UC-grafted leather with PFDT.

Covalent surface modification of CL and VL was achieved via esterification reaction between hydroxyl moieties of the hydroxyproline amino acids which are part of the collagen backbone and acyl chloride moiety of UC as shown in the experimental section. The presence of free hydroxyl groups on the surface of CL and VL was confirmed by ATR-IR measurements that exhibited a broad peak between 3100 and 3400 cm^−1^ for both types of leather (Figure 1 and Figure S1). The esterification reaction between fish leathers and UC was carried out using TEA, which served as an HCl scavenger and was released during the reaction (Scheme 2A). Water contact angle measurements revealed a successful covalent modification with aliphatic small molecules as the hydrophilic unmodified CL and VL were transformed into hydrophobic ones with a WCA of 126° ± 6 detected for both types of fish leather (Table 2, Figure 3 and Figure S2). UC treated fish leather exhibited WCA values that were only slightly lower than the ones obtained for UC grafted cotton fabrics (132° ± 2) reported in our previous study [48]. The difference in the WCA values achieved for fish leather and cotton fabric can be attributed to the dissimilarity in the chemical nature of each surface. While the surface of cotton fabric contains chemically reactive primary hydroxyl groups in each repeating unit of the cellulose, these groups are less abundant and periodic on the surface of the fish leather due to the structure of the collagen backbone and the effect of the tanning process.

As previously shown by our group and others, the thiol-ene click reaction can be utilized for a wide range of thiol and alkene containing-materials with short reaction times and high yields [49,57,58]. We thus examined covalent binding of PFDT to the UC-grafted CL and VL leather surfaces by placing the leathers into a chloroform solution containing DMPA as a photo-initiator, followed by irradiation at 365 nm for 20 min on the scale side of the leather (Scheme 2B).

XPS measurements were conducted to validate covalent modification with UC and PFDT molecules. Table 3 shows the atomic composition of neat and modified VL and CL, respectively. The O/C ratio could provide clear indication to a successful grafting reaction. First, a significant difference was observed in the O/C ratios between neat-CL and VL leathers which were 0.42 and 0.21, respectively. This difference in the O/C ratio can be attributed to the different reagents used in the tanning procedures for each type of leather. In contrast, both UC treated CL and VL leathers exhibited similar O/C ratios of about 0.3, corresponding to the attachment of aliphatic chain that contains eleven carbon atoms and indicating that a uniform molecular coating was achieved for both CL and VL.

**Figure 4:**
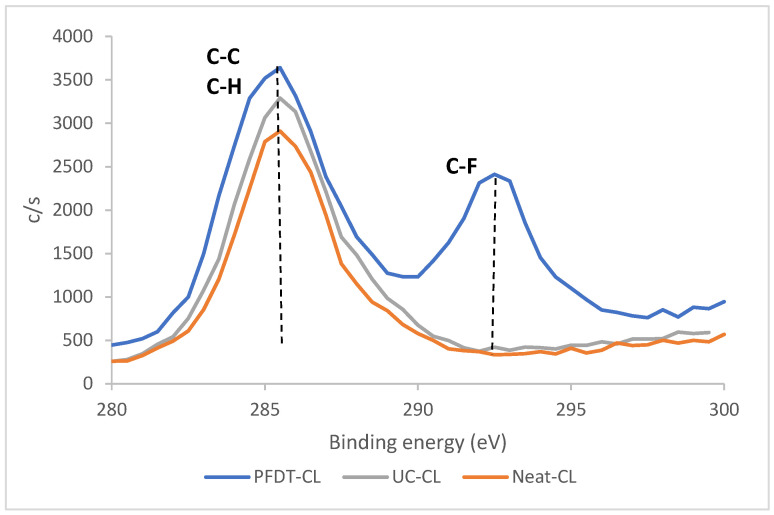
C1s comparison (low resolution XPS) of neat and modified CL.

Furthermore, both PFDT modified VL and CL showed significant amounts of fluorine atoms for the PFDT-modified leather with at least 20% of the surface composition even after excessive Soxhlet wash, as well as the substantial peak at 292 eV in binding energy spectra, which was ascribed to F-C bond (Figure 4 for CL and Figure S3 for VL). These results confirm a chemical grafting to the leather surface, which occurred between the double bond of UC and the thiol group of PFDT. Moreover, the amount of chromium detected on the surface for each modification stage was reduced from 2.09% for the neat to 1.47% for the UC- modified and to 0.56% for the PFDT-modified CL leathers. These results support the formation of thin coating layers on the surface of the leather due to the covalent binding of small functional molecules.

The covalent surface modification with PFDT resulted in further increase in surface hydrophobicity as demonstrated by the increase in water contact angle from 126° for UC-modified to 134° for PFDT-modified leathers. 

### 3.3. Amphiphobic Coating

**Scheme 3:**
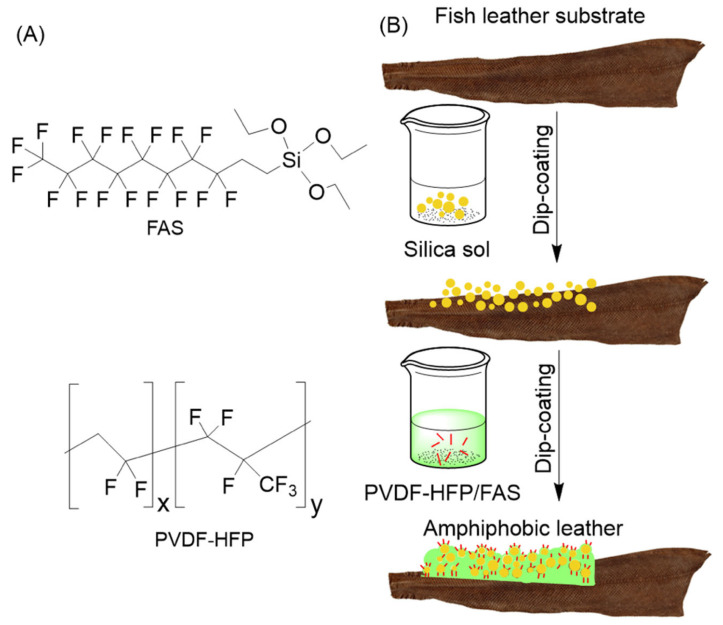
(**A**) Molecular structures of FAS and PVDF-HFP. (**B**) Schematic representation of the process for fabrication of amphiphobic coating.

The fabrication of CL and VL with amphiphobic coating is schematically represented in Scheme 3B, whereas Scheme 3A depicts the molecular structures of PVDF-HFP and FAS. Two coating solutions were sequentially applied to the fish leather via dip-coating method as detailed in the experimental section. First, CL and VL were immersed in FSN solution that was formed by co-hydrolyzing TEOS and FAS in ethanol, intending to increase the surface roughness of the leather, followed by dipping it into the PVDF-HFP/FAS solution to decrease the surface energy [59]. We anticipated that a combination of the two coatings would create a repellent surface for both water and oil.

**Figure 5:**
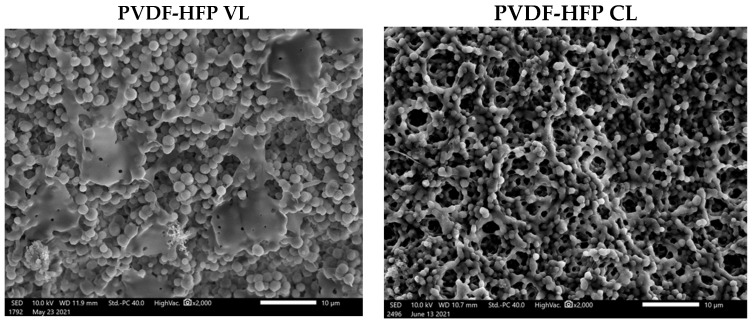
SEM photographs of CL and VL after PVDF-HFP coating.

Analysis of the surface morphology of the treated leathers by the SEM revealed that the incorporation of the amphiphobic coating leads to significant changes for both types of fish leather, transforming the fibrous structure observed prior to the modification into a polymer composite coating containing interconnected spherical particles (Figure 5). Although a similar coating process was used for both VL and CL, the particle size of the coatings was slightly different and exhibited average values of 1.78 ± 0.21 µm and 1.25 ± 0.14 µm, respectively (Figure S6).

AFM was further used to examine the topography and the changes in the surface roughness of neat and coated leathers. The AFM images of the neat leather showed the natural topography of the leather with only minor deviations in the height resulting from the random arrangement of the collagen fibers providing micro-scale roughness (Figure S4). In contrast, the AFM topography images of the coated leathers showed significant height differences due to the spherical structure of the particles providing nanoscale roughness. The root means square roughness of both PVDF-HFP coated VL and CL was dramatically increased from 48 to 534 and from 71 to 254, respectively (Figure S4). The difference in the surface roughness between VL and CL can be attributed to the difference in the particle size of the coatings, which displayed larger values for the VL in comparison with the CL according to the SEM analysis. A combination of the nanoscale roughness due to the amphiphobic coating with a macroscale roughness inherently found in the leather fibril structure, was most likely the reason behind the considerable increase in the water- and-oil-repellence properties of the coated leathers. Furthermore, a non-stainability effect was observed for the coated leather as the water and oil droplets were easily wiped without leaving any trace on the coated leather.

**Figure 6:**
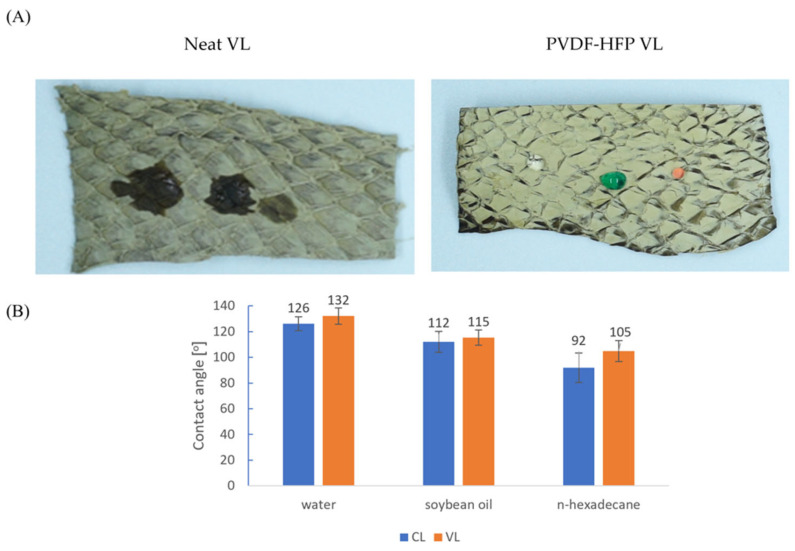
(**A**) Photographs (samples of 1 × 3 cm) of VL before and after PVDF-HFP modification taken using drops of water (dyed red) soybean oil (dyed green) and n-hexadecane. (**B**) Contact angle values of PVDF-HFP coated leathers.

The amphiphobic nature of the coated VL is presented in Figure 6A using water (dyed red), soybean oil (dyed green), and n-hexadecane. Contact angles were measured to quantify the ability of the coated leathers to repel water, soybean oil, and n-hexadecane (Figure 6B). The modified CL exhibited contact angles of 126°, 112°, and 96° and VL showed 132°, 115°, and 105° for water, soybean oil, and n-hexadecane, respectively. These results demonstrate that fish leather with excellent repellent properties for polar and non-polar liquids can be realized by the introduction of amphiphobic coating containing a low surface energy polymer (PVDF-HFP) and silica nanoparticles for increasing the surface roughness.

Water vapor permeability of materials designated for wearable applications is an important parameter that influences the comfort of the clothing [47]. Therefore, WVTR measurements were conducted to compare the effect of hydrophobic and amphiphobic surface modifications on the breathability of the fish leather. The results showed that neat CL exhibited a water vapor permeability value of 22.2 g/(m^2^day), while the corresponding values for the UC-modified and PVDF-HFP coated leathers were 2.3 and 60.9 g/(m^2^day), respectively. The reduction in water vapor permeability observed for the UC grafted fish leather in comparison with the unmodified one is most likely due to the increased hydrophobicity of the UC-coated leather. In contrast, the PVDF-HFP coating showed a significant increase in WVTR, allowing the coated leather to “breathe”. This outstanding property can be associated with the porosity that was achieved on the surface by silica nanoparticles that allowed a more efficient penetration of water molecules [9,10].

## 4. Conclusions

Three efficient finishing treatments have been successfully developed on vegetable and chromium-tanned salmon leather. Despite the differences in the tanning methods used for the preparation of CL and VL, no significant variations were found in their ability to undergo the coating procedures. PANI-coated fish leathers showed efficient surface electrical conductivity with a reduction of five to six orders of magnitude in surface resistivity in comparison with the uncoated leathers. In addition, UC surface grafting resulted in a dramatic change in the wettability of the fish leathers which were transformed from hydrophilic to hydrophobic ones. Furthermore, the ability to use double bond end-groups present on the surface of UC-grafted leathers for further chemical functionalization was demonstrated by covalent attachment of PFDT using thiol-ene click reaction conditions. Finally, PVDF-HFP/silica nanoparticles coated fish leathers showed amphiphobic and non-staining properties with considerably improved water vapor permeability, making this type of leather a promising candidate for applications in the fashion industry. Overall, the developed surface modifications significantly improved the functionality of the fish leather, and we hope this research can broaden its uses and applications in various fields.

## Figures and Tables

**Table 1:** Surface resistivity of neat and PANI coated VL and CL.

Leather Type	$\mathrm{Scale}\;\mathrm{Side}\;(\mathrm{Log}\;(\frac{Ω}{square})$)	$\mathrm{Flesh}\;\mathrm{Side}\;(\mathrm{Log}\;(\frac{Ω}{square})$)
Neat CL	11.4 ± 0.4	11.8 ± 0.3
PANI-CL	5.5 ± 0.4	5.2 ± 0.4
Neat VL	11.7 ± 0.2	11.7 ± 0.1
PANI-VL	6.8 ± 0.3	5.2 ± 0.2

**Table 2:** Water contact angle values of neat and modified leather.

Leather	Neat	UC	PFDT
VL	0	126° ± 6	134° ± 4
CL	0	126° ± 6	134° ± 4

**Table 3:** Surface atomic composition of neat and modified VL and CL.

% Atomic Composition						
	C	O	N	F	Cr	S
NEAT-CL	62.26	26.12	6.2	-	2.09	1.54
UC-CL	68.77	20.87	6.17	0.36	1.47	1.36
PFDT-CL	42.89	13.23	2.12	36.46	0.56	2.25
NEAT-VL	78.45	16.46	0.93	-	-	1.35
UC-VL	68.58	22.17	5.43	-	-	1.23
PFDT-VL	51.42	18.72	4.29	19.58	-	1.81

## Data Availability

The data that support the findings of this study are available on request from the corresponding author.

## Supplementary Materials

The following supporting information can be downloaded at: https://www.mdpi.com/article/10.3390/polym15193904/s1, Figure S1. FTIR results neat and PANI coated VL; Figure S2. Photographs of VL before and after UC treatment taken using drops of water (dyed red); Figure S3. C1s comparison (low resolution) of neat and modified VL; Figure S4. AFM topography photographs demonstrating the change in roughness between neat and coated leather; Figure S5. SEM photographs after measurement of fibril diameters of neat and PANI coated CL; Table S1. Collagen fibers diameter measurement of neat and PANI coated CL; Figure S6. SEM photographs showing the morphology of CL and VL after PVDF-HFP coating.
