# Supplementary material for: Increasing Functionality of Fish Leather by Chemical Surface Modifications
Source: Polymers (Basel). 2023 Sep 27;15(19):3904. doi: 10.3390/polym15193904 (PMC10574862; doi:10.3390/polym15193904)
Supplement: Supplementary file 1 [file polymers-15-03904-s001.zip › polymers-2559558-supplementary.pdf]

Supporting Information

Increasing Functionality of Fish Leather by Chemical Surface Modifications

Achiad Zilberfarb, Gali Cohen and Elizabeth Amir*

Department of Polymer Materials Engineering, Shenkar College, Ramat-Gan, Israel

Correspondence: eamir@shenkar.ac.il

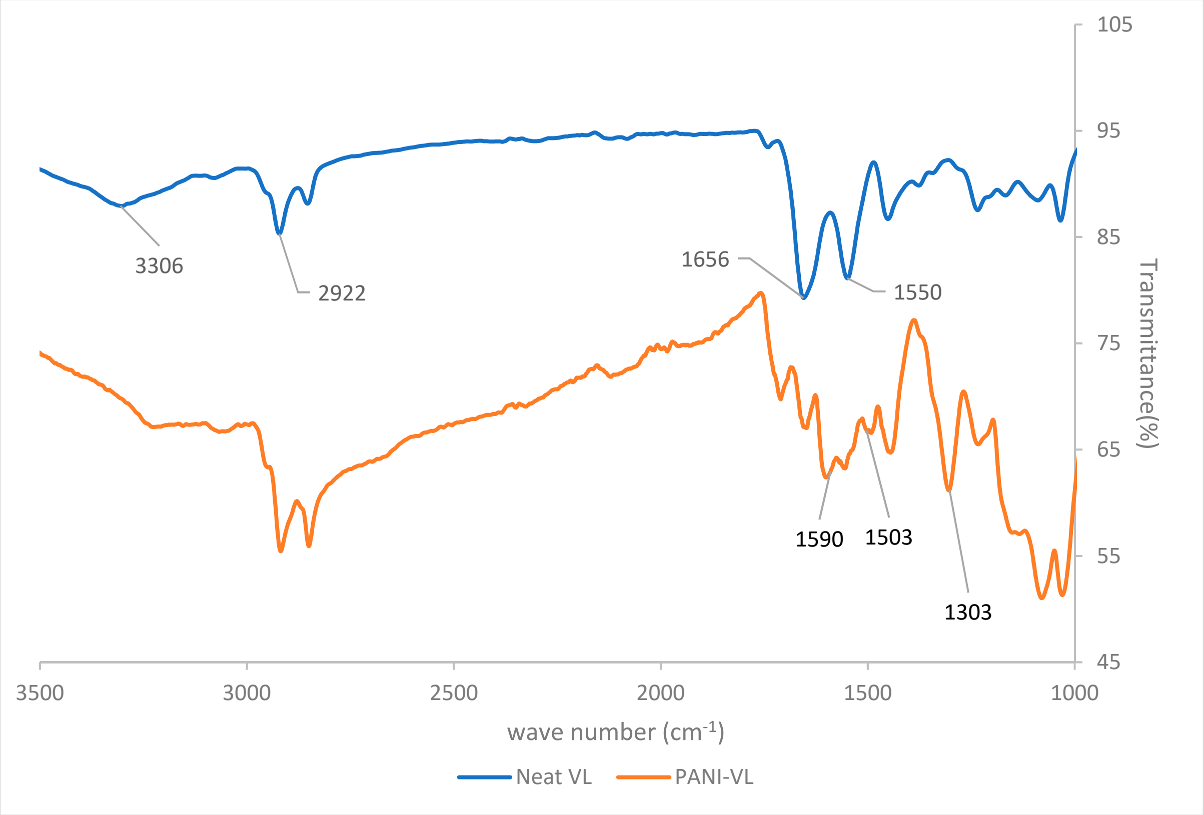

Figure S1. FTIR results neat and PANI coated VL.

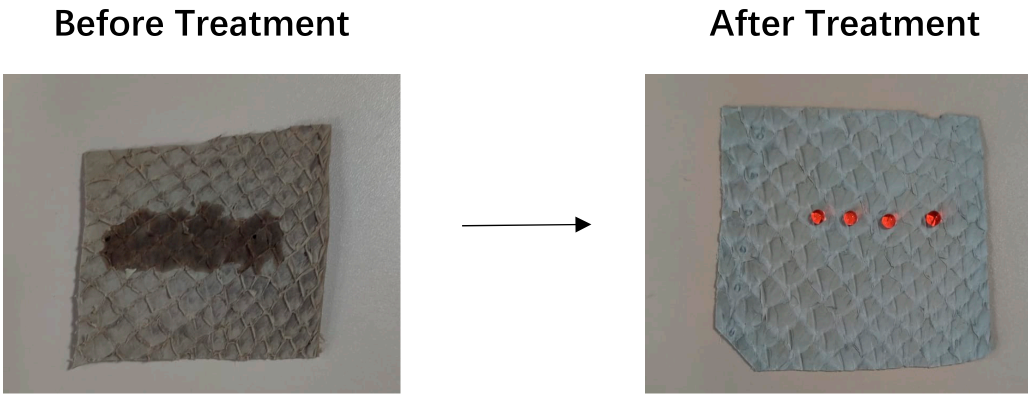

Figure S2. Photographs of VL before and after UC treatment taken using drops of water (dyed red).

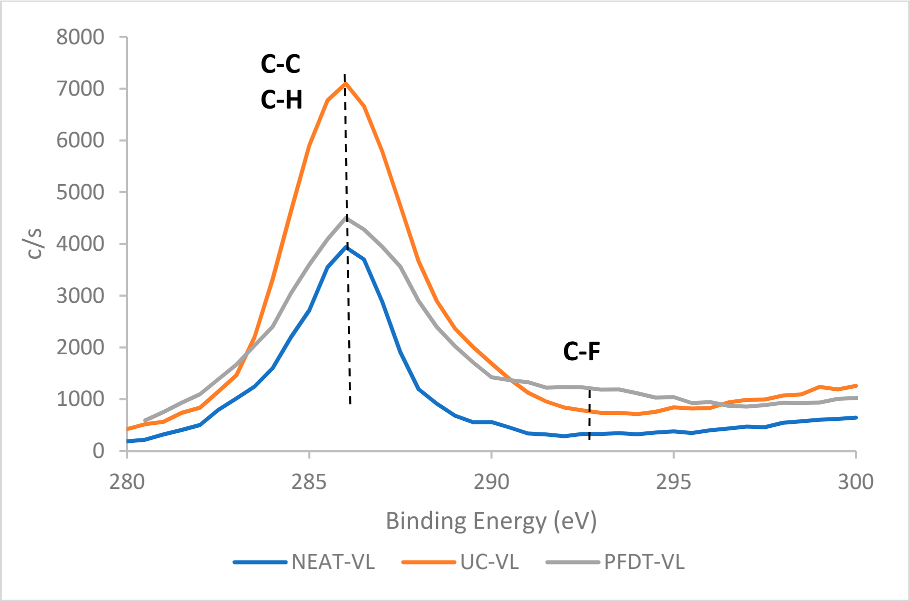

Figure S3. C1s comparison (low resolution) of neat and modified VL.

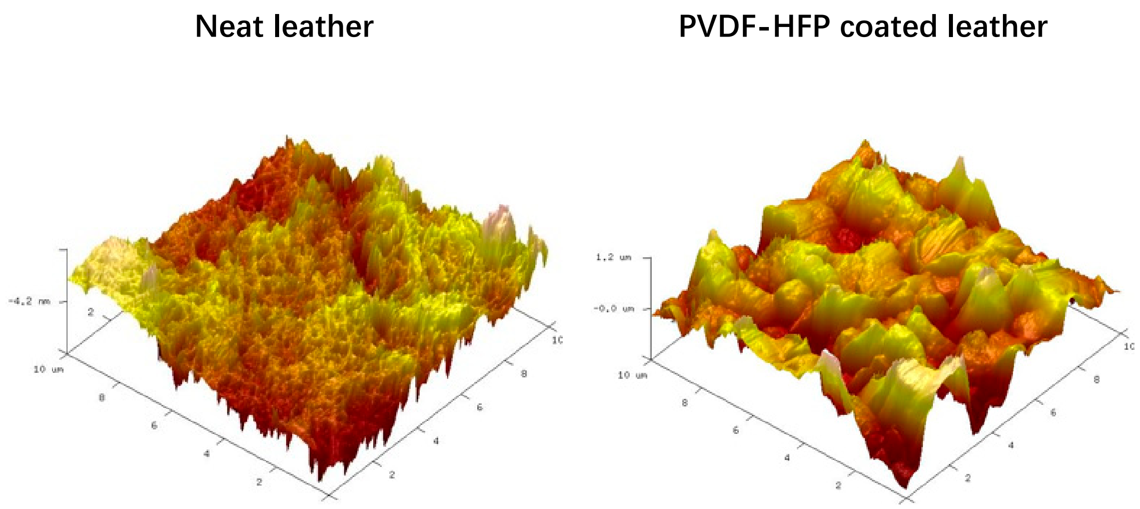

Figure S4. AFM topography photographs demonstrating the change in roughness between neat and coated leather.

Table S1. Collagen fibers diameter measurement of neat and PANI coated CL.

Sample	Neat CL (µm)	PANI CL (µm)
1	0.285	0.235
2	0.202	0.446
3	0.179	0.331
4	0.218	0.304
5	0.135	0.341
6	0.195	0.206
7	0.169	0.148
8	0.19	0.215
9	0.151	0.39
10	0.176	0.197
**AVG**	**0.19**	**0.281**
**SD**	**0.041**	**0.096**

Neat CL **PANI CL**

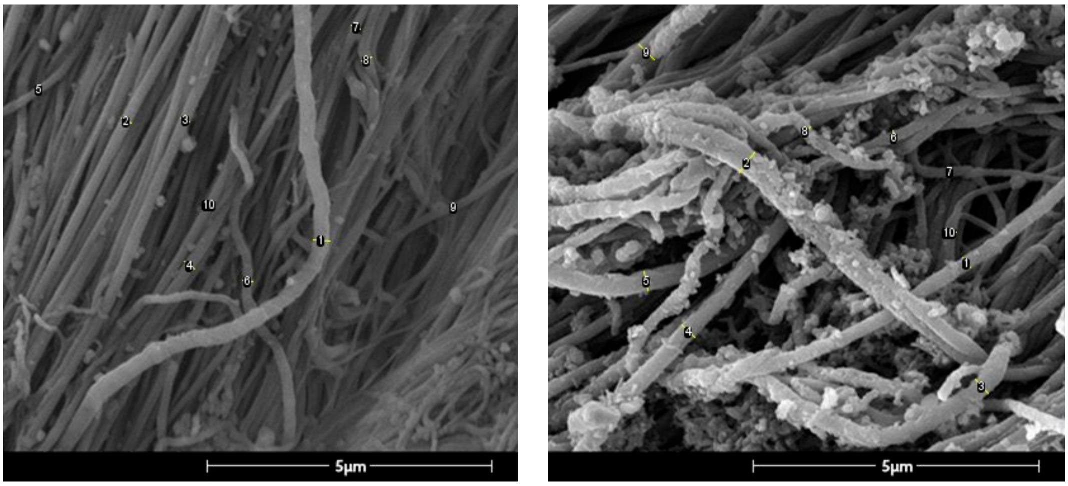

Figure S5. SEM photographs showing measurements of fibril diameters of neat and PANI coated CL.

PVDF-HFP VL **PVDF-HFP CL**

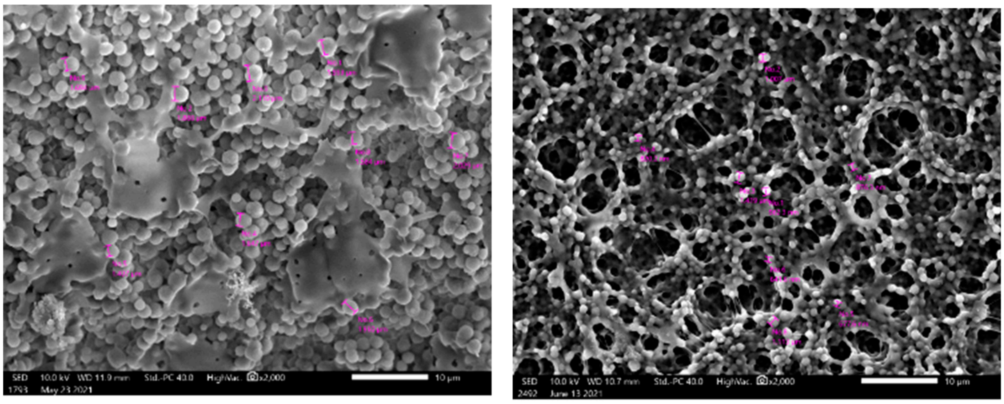

Figure S6. SEM photographs showing the morphology of CL and VL after PVDF-HFP coating.
